# COVID-19: Risk Stratification of Healthcare Workers in the Eastern Province of Saudi Arabia and Their Knowledge, Attitude, and Fears

**DOI:** 10.7759/cureus.19652

**Published:** 2021-11-17

**Authors:** Rabia Latif, Sara Alali, Rasha AlNujaidi, Leyan Alotaibi, Nada Alghamdi, Maha Alblaies

**Affiliations:** 1 Physiology, Imam Abdulrahman Bin Faisal University, Dammam, SAU; 2 Infectious Diseases, Imam Abdulrahman Bin Faisal University, Dammam, SAU

**Keywords:** fear, covid-19, attitude, knowledge, healthcare workers

## Abstract

Introduction

With the expeditious spread of coronavirus disease 2019 (COVID-19), healthcare workers have undoubtedly faced a higher risk of contracting the disease compared to the general public. This study aimed to stratify the risk of coronavirus disease 2019 infection among healthcare workers in the Eastern province of Saudi Arabia and shed light on their level of knowledge, attitude, and fear towards the disease.

Methods

A quantitative cross-sectional study, involving 978 Arabic and English-speaking healthcare workers, was conducted using a self-administered online questionnaire. The knowledge, attitude, and fear scales were developed by researchers using the most updated information regarding coronavirus disease 2019. The Objective Risk Stratification tool developed in the United Kingdom was used to measure the risk level of contracting coronavirus disease 2019. Collected data were analyzed and interpreted using the Statistical Package for Social Sciences software.

Results

Out of the 978 participants, 63.1% were female, 74.6% were 20-39 years old, 86.9% were Saudis, and 31.3% worked as physicians. The most common health-related risk factors for severe coronavirus disease 2019 among the study participants were smoking (23.4%), sickle cell trait (22.8%), and asthma (21.2%). The risk of contracting coronavirus disease 2019 was found to be low in 87.2% of participants, with those significantly at higher risk being male, non-Saudis, black Africans, and 70-79 years old. The knowledge level was found to be high among 54.7% of participants, with significantly higher levels being reported among females, non-Saudis, and participants who were either physicians or pharmacists. The most commonly cited source of knowledge was the Saudi Ministry of Health (82%). Participants largely demonstrated a positive attitude towards the disease (53.9%), particularly those working as physicians and in the governmental sector. The majority of participants (54.4%) were found to have a high level of fear toward the disease, with significantly higher levels being reported among females, 30-39 years old, and those who were either nurses or pharmacists.

Conclusion

The present study demonstrated significant sociodemographic variability among healthcare workers in the Eastern province, with respect to their risk of contracting coronavirus disease 2019 and their levels of knowledge, attitude, and fear toward the disease.

## Introduction

Coronavirus disease 2019 (COVID-19) is an infection of the respiratory system [1]. The disease has spread rapidly worldwide, starting from Wuhan, China, in December of 2019. It has unfortunately succeeded at impending the lives of a large number of individuals, with 111,270,860 confirmed cases of COVID-19, including 2,466,639 reported deaths worldwide, as of February 23, 2021 [2]. Similarly, the Kingdom of Saudi Arabia (KSA) has also been greatly affected. With an estimated population of 34,813,871 citizens as of mid-year 2020, over 375,333 cases have been reported locally, including 6,466 confirmed deaths, as of February 23, 2021 [2].

Consequently, on January 30, 2020, the World Health Organization (WHO) declared the COVID-19 outbreak as a global health crisis; and on March 11, 2020, it was proclaimed as a pandemic [2]. Correspondingly, many countries around the world have responded by implementing vigorous infection control regulations, aiming for the mitigation of the disease, and KSA was no exception. Nevertheless, the spread of infection is highly dependent on the knowledge and attitude toward the disease, in which both factors can influence individuals’ readiness and willingness to follow appropriate infection control guidelines. Given the fact that healthcare workers (HCWs) are considered linchpins of controlling COVID-19, as well as managing it, HCWs’ infection by severe acute respiratory syndrome coronavirus 2 (SARS-CoV-2) is an extremely critical concern that needs to be taken into great consideration. With HCWs being the major frontline workforce, they are at higher risk of being infected, as evidenced by the over 1000 cases of COVID-19 deaths reported among HCWs globally by May 14, 2020 [3-4]. Accordingly, numerous organizations, such as National Health Service (NHS) Employers and the Faculty of Occupational Medicine, have accentuated the requisite for pragmatic risk stratification of COVID-19 infection among HCWs; thereby decreasing the risk for all HCWs [5]. Fear may also contribute to HCWs' attitude towards COVID-19, especially given the obscure and undetermined features of the disease. Thus, the identification of knowledge, attitude, and fear levels among HCWs can be utilized to create a roadmap for improving and reforming regulations in the clinical setting, thereby contributing to the deceleration of infection spread. Similarly, assessing and stratifying the risk of COVID-19 among HCWs is paramount in attainting targeted and suitable infection control and prevention recommendations, as well as restructuring clinical duties based on risk.

A study in the United Kingdom has been designed to formulate an empiric risk assessment tool known as the Objective Risk Stratification (ORS) tool. Unfortunately, there are no current studies designed to measure the risk of COVID-19 infection among HCWs in KSA [5]. Multiple studies have been conducted, to measure the level of knowledge and attitude of COVID-19 among HCWs; however, most have been published before or very shortly after, the declaration of the COVID-19 outbreak as a pandemic. Hence, the mean level of knowledge among HCWs, currently, is expected to have changed exponentially from the earlier stages of the pandemic. Additionally, the sample representing HCWs in most published studies has been commonly limited to the inclusion of physicians, nurses, and one or two other healthcare personnel. Thereby, disregarding many members of the healthcare system, which may contribute to a variation in knowledge and attitude levels among HCWs [3,6-9]. As for the element of fear, though taken into perspective in some studies, it was not appraised accurately, for the surveys lacked a variety of circumstances in which COVID-19 could generate fear. The studies have also neglected to correlate the levels of fear to the variations in knowledge, attitude, and practices (KAP) of COVID-19 among HCWs. Furthermore, due to the augmenting rate of COVID-19 in KSA, the evaluation of knowledge, attitude, and fear of COVID-19 among HCWs in the region are needed, by which one study did assess the aforementioned elements; however, was limited by the fact that it was conducted before the emergence of the first case of COVID-19 in KSA, which could have greatly skewed the reported knowledge and attitude levels among HCWs who have yet to encounter any COVID-19 case head-on. Another limitation of the study is its restriction of the sample to one tertiary hospital, which may eclipse potential existing variations in different centers. Additionally, another study assessed the knowledge, the attitude of HCWs about COVID-19 in Saudi Arabia but did not assess the level of fear among HCWs or correlate it with their knowledge and attitude levels [10]. Given the limitations of all aforementioned studies, this study aimed to include a more diverse sample of HCWs and assess their levels of knowledge, attitude, and fear towards COVID-19 during a period of increased COVID-19 cases.

This present study aimed to stratify the risk of COVID-19 infection among HCWs, within the Eastern province of Saudi Arabia, as well as quantify their levels of knowledge, attitude, and fear towards COVID-19. It also intended to identify any significant sociodemographic variability or correlations between the aforementioned levels.

## Materials and methods

The study design was in the form of a cross-sectional study, targeting HCWs in the Eastern province of Saudi Arabia and was conducted from December 2020 to February 2021. Participants were recruited through snowball sampling and the sample size was calculated as 384 using the EpiInfoTM application version 5.5.2, where the response rate was set at 50%, confidence interval at 95%, and a margin of error at 5%. The inclusion criteria were HCWs, of different nationalities, working in either public or private healthcare sectors within the Eastern province. The main exclusion criteria were (1) non-Arabic or non-English speaking HCWs an­­d (2) HCWs working in other provinces within Saudi Arabia.

A self-administered online questionnaire was developed in both the Arabic and English languages using the most recently available information about COVID-19 from the Center for Disease Control (CDC) and UpToDate. It consisted of four sections, including demographics, knowledge scale, attitude scale, and fear scale. 

The demographics were composed of seven items, including sex, age, nationality, ethnicity working sector, role in healthcare, and health-related risk factors for severe COVID-19. Sex, age, ethnicity, and health-related risk factors for severe COVID-19 were used to stratify healthcare workers’ risk of COVID-19 infection based on a pre-validated objective risk stratification (ORS) tool [5].

The knowledge scale was inspired by a 2020 study conducted in Nepal and was composed of 61 items/questions, and responses were scored as one for correct answers, zero for incorrect, or “I don’t know” answers [11].

The attitude scale was Inspired by a 2020 study conducted in Nepal and was composed of six items/questions, by which responses were scored from one-five corresponding to the Likert’s agreement scale (1-5) [11].

The fear scale was inspired by the Fear of COVID-19 Scale (FCV-19S) and was composed of eight items/questions, by which responses scored from 1-5 corresponding to the Likert’s agreement scale (1-5) [12].

The study variables and measurements are demonstrated in Table 1.

**Table 1:** Study variables and measurements

Variable Type		Scale of Measurement
Independent	Sex	Nominal categorical: female, male
	Age	Ordinal categorical: 20-29 30-39 40-49 50-59 60-69 70-79
	Nationality	Nominal categorical: Saudi, non-Saudi
	Working sector	Nominal categorical: governmental, private
	Role in health care	Nominal categorical: Physician, Dentist, Nurse, Pharmacist, Health-associated professionals (i.e., dietician, paramedic, patient care assistant, physiotherapist, technician) Health management and supportive personnel (i.e., administrator, health educator, psychologist, quality control, receptionist, social worker)
	Health-related risk factors for COVID-19	Nominal categorical: present, absent
	Source of Knowledge	Nominal categorical: Center for Disease Control (CDC), Ministry of Health (MOH), Practice in a healthcare setting, Social media, World Health Organization (WHO)
Dependent	Knowledge level	Nominal categorical: Low level: scores below the median score; High level: scores equal to or above the median score
	Attitude level	Nominal categorical: Positive attitude: scores equal to or above median score; Negative attitude: scores below the median score
	Fear level	Nominal categorical: Low level: scores below the median score; High level: scores equal to or above the median score
	Risk level	Ordinal categorical: Low risk: less than 3; Medium risk: 3-5; High risk: equal to or above 6 [5]

After its development, both versions of the questionnaire were uploaded onto Google Forms and re-evaluated by the researchers for any possible errors or discrepancies. A pilot study, involving 20 participants, was then conducted using both versions of the online questionnaire. Pilot study data were analyzed using the Statistical Package for Social Sciences (SPSS) software, where the internal validity of the scales employed was ensured using Cronbach’s Alpha. Accordingly, no differences between the two versions were noted and no modifications were made to the questionnaire. The questionnaire was then disseminated using multiple social media platforms including WhatsApp, Twitter, and Telegram.

The collected data were manually input by the researchers into SPSS software version 26.0.0.0 (IBM Corp., Armonk, NY) with the level of significance set at below 0.05. During input, the data were subjected to filtration, whereby 378 participants were excluded from the study due to not meeting the inclusion criteria. The remaining data that met the inclusion criteria were tested using the Shapiro-Wilk test and were found to not have a normal distribution. Subsequently, the median and interquartile ranges were used as a measure of central tendency instead of the mean. The data collected from the three scales mentioned above was then subjected to reliability analysis using Cronbach’s Alpha, which revealed a high level of internal consistency (α = 0.634 - 0.854). Data regarding participants’ role in healthcare were grouped according to the WHO classification system of healthcare workers, as shown in the variables table above [13]. Simple frequency tables were used to illustrate the demographic characteristics of study participants, health-related risk factors for severe COVID-19, the pattern of responses for each question listed in the questionnaire, and sources of knowledge. The median score for each of the three scales (knowledge, attitude, and fear) was calculated and used as a cutoff point to stratify participants into different levels, as clarified above in the variables table. The data collected from the demographics section were used to stratify the participants into three risk levels, shown in the variables table above, according to the previously mentioned ORS scale. The chi-square test and Fisher’s test were used to analyze the differences in proportion between the knowledge, attitude, fear, risk levels, and demographic variables. Cramer’s V (φc) was used as an in-effect size to determine the magnitude of the association where 0.10 was considered as a small effect, .30 as a medium effect, and .50 as a large effect as reported by Cohen (1988) [14]. The Spearman’s rank correlation coefficient was used to evaluate the interrelationship between knowledge, attitude, fear, and risk scores.

Regarding ethical considerations, the current study was conducted after obtaining ethical approval (IRB-UGS-2020-01-318) from the institutional review board (IRB) at Imam Abdulrahman Bin Faisal University. The anonymity and confidentiality of participants’ responses were guaranteed, and participation in the study was strictly voluntary. Completion and submission of the questionnaire were considered as informed consent.

## Results

Sociodemographic characteristics of study participants

A total of 978 participants completed the survey and met the eligibility criteria set by researchers. The majority of the participants were female (63.1%) and 20-39 years old (74.6%). More than three-quarters of the respondents were Saudi (86.9%), originally from the Middle East (82.5%), and worked in governmental institutions (80.8%). Out of all participants, 31.3% were physicians, 21.9% were health-associated professionals, and 19.5% were nurses. Furthermore, 36.7% of healthcare workers reported that they have health-related risk factors for severe COVID-19 while 63.3% did not, as shown in Table 2.

**Table 2:** Sociodemographic characteristics of study participants

Demographic Information		Number (%)
Sex	Female	617 (63.1)
	Male	361 (36.9)
Age Group (Years)	20-29	414 (42.3)
	30-39	316 (32.3)
	40-49	171 (17.5)
	50-59	59 (6)
	60-69	16 (1.6)
	70-79	2 (0.2)
Nationality	Non-Saudi	128 (13.1)
	Saudi	850 (86.9)
Ethnicity	Black African descent	13 (1.3)
	Caucasian	22 (2.2)
	Indian Asian	136 (13.9)
	Middle Eastern	807 (82.5)
Working Sector	Governmental	790 (80.8)
	Private	188 (19.2)
Role in Healthcare	Dentist	63 (6.4)
	Health associate professionals	214 (21.9)
	Health management and support personnel	143 (14.6)
	Nurse	191 (19.5)
	Pharmacist	61 (6.2)
	Physician	306 (31.3)
Health-Related Risk Factors	Absent	619 (63.3)
	Present	359 (36.7)

**Figure 1:**
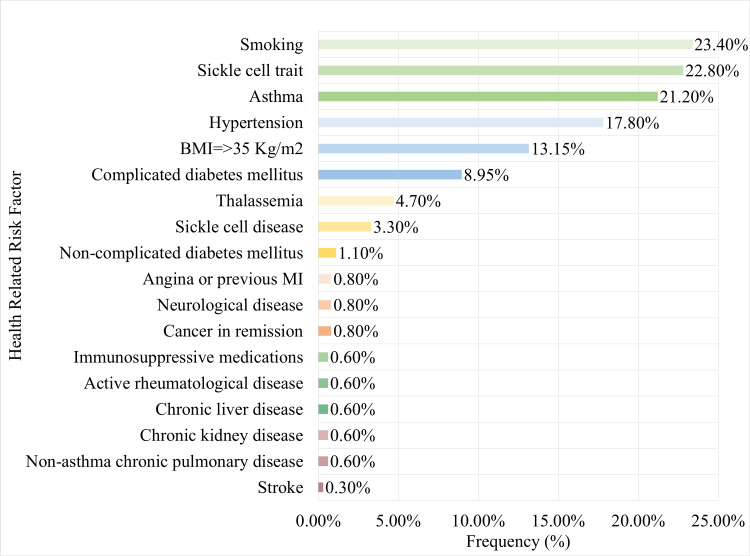
Frequency of health-related risk factors for severe COVID-19 infection among study participants BMI: body mass index

For those who reported the presence of health-related risk factors for severe COVID-19, the risk factors were arranged in descending order from the highest to the lowest percent. Importantly, smoking (23.4%) and sickle cell trait (22.8%) were the major risk factors reported among the study participants as shown in Figure 1.

Reliability analysis of the study instrument

Reliability analysis was carried out utilizing the three scales (COVID-19 knowledge, attitude, fear), which were comprised of 61.6 and eight items, respectively. Based on the calculated sample, Cronbach's alpha showed a high level of internal consistency ranging between α=0.634 to 0.854 (Table 3).

**Table 3:** Descriptive statistics and Cronbach's Alpha for COVID-19 knowledge, attitude, and fear scales Min: minimum; Max: maximum; IQR: interquartile range

Scale	Number of Items	Range (Min-Max)	Median (IQR)	Cronbach Alpha
Knowledge	61	1-61	49 (6)	.854
Attitude	6	6-30	29 (3)	.634
Fear	8	8-40	23 (9)	.652

Knowledge, attitude, and fear response patterns

Tables 4-6 outline the study participants’ response pattern for each of the three scales: knowledge, attitude, and fear during COVID-19, respectively.

**Table 4:** Study participants’ response pattern to questions related to COVID-19 knowledge PPE: personal protective equipment

Section	Statement	Number (%)		
		Correct	Incorrect	Total
COVID-19 Virology	COVID-19 is SARS-CoV-2	646 (66)	332 (34)	978 (100)
	The time between acquiring the infection till the onset of symptoms is 14 days	770 (79)	208 (21)	978 (100)
COVID-19 Epidemiology and Transmission	COVID-19 was first identified in Wuhan China	965 (99)	13 (1)	978 (100)
	COVID-19 is a pandemic	972 (99)	6 (1)	978 (100)
	COVID-19 does not spread from one person to another	876 (90)	102 (10)	978 (100)
	COVID-19 spreads through respiratory droplet	949 (97)	29 (3)	978 (100)
	The precise interval during which an infected individual can infect others is uncertain	729 (75)	249 (25)	978 (100)
	The virus can spread even if the infected individual is asymptomatic	889 (91)	89 (9)	978 (100)
	Outdoor settings do not put you at risk in case of contact with an infected individual	742 (76)	236 (24)	978 (100)
	Touching surfaces contaminated with the viral droplet does not aid in transmission	852 (87)	126 (13)	978 (100)
	There is evidence towards animals (including domesticated animals) being considered a major source of transmission	373 (38)	605 (62)	978 (100)
	The duration of protective immunity after COVID-19 infection is not known	738 (75)	240 (25)	978 (100)
The following condition is considered a risk factor for severe COVID-19	Cardiovascular diseases	907 (93)	71 (7)	978 (100)
	Chronic kidney disease	798 (82)	180 (18)	978 (100)
	Chronic respiratory disease	946 (97)	32 (3)	978 (100)
	Diabetes mellitus	863 (88)	115 (12)	978 (100)
	Eczema	602 (62)	376 (38)	978 (100)
	Eye refractive error	731 (75)	247 (25)	978 (100)
	Gastroesophageal reflux disease (GERD)	494 (51)	484 (49)	978 (100)
	Hypertension	838 (86)	140 (14)	978 (100)
	Obesity (BMI ≥30 kg/m2)	820 (84)	158 (16)	978 (100)
	Old age ≥ 65 years	926 (95)	52 (5)	978 (100)
	Malignancy	798 (82)	180 (18)	978 (100)
	Migraine	535 (55)	443 (45)	978 (100)
	Pregnancy	639 (65)	339 (35)	978 (100)
	Sickle cell disease or trait	647 (66)	331 (34)	978 (100)
	Smoking	812 (83)	166 (17)	978 (100)
Clinical features of COVID-19	COVID-19 infected individuals can be asymptomatic	968 (99)	10 (1)	978 (100)
	COVID-19 infection is generally life-threatening and associated with high mortality	245 (25)	733 (75)	978 (100)
	Pneumonia is the most serious manifestation	892 (91)	86 (9%)	978 (100)
	Acute respiratory distress syndrome is the most serious complication	936 (96)	42 (4)	978 (100)
	Recovery course is certain and known amongst all infected individuals	632 (65)	346 (35)	978 (100)
The following is considered one of the symptoms of COVID-19	Abdominal pain	279 (29)	699 (71)	978 (100)
	Cough	965 (99)	13 (1)	978 (100)
	Diarrhea	870 (89)	108 (11)	978 (100)
	Dyspnea	956 (97)	22 (3)	978 (100)
	Fever	971 (99)	7 (1)	978 (100)
	Headache	938 (96)	40 (4)	978 (100)
	Loss of smell or taste sensation	966 (99)	12 (1)	978 (100)
	Myalgia	903 (92)	75 (8)	978 (100)
	Nausea and vomiting	782 (80)	196 (20)	978 (100)
	Rhinorrhea	240 (25)	738 (75)	978 (100)
	Sore throat	882 (90)	96 (10)	978 (100)
The following is an acceptable sample for RT-PCR	Nasopharyngeal swab	941 (96)	37 (4)	978 (100)
	Oropharyngeal swab	872 (89)	106 (11)	978 (100)
	Nasal or nasopharyngeal wash/aspirate	301 (31)	677 (69)	978 (100)
	Nasal swab specimen from both anterior nares	544 (56)	434 (44)	978 (100)
COVID-19 management	All individuals testing positive need to be hospitalized	847 (87)	131 (13)	978 (100)
	All non-hospitalized patients need to be self-isolated for the anticipated duration	922 (94)	56 (6)	978 (100)
	For non-hospitalized patients supportive therapy (i.e. antipyretic, hydration, rest) is the mainstay of treatment	897 (92)	81 (8)	978 (100)
	There is a definite medication to eradicate COVID-19	761 (78)	217 (22)	978 (100)
COVID-19 prevention	Wearing masks in public and social distancing can break the chain of infection	971 (99)	7 (1)	978 (100)
	Hand washing after touching contaminated surfaces does not reduce the risk of infection	488 (50)	490 (50)	978 (100)
	Hand washing should be at least for 20 seconds	909 (93)	69 (7)	978 (100)
	There has been a well-established vaccine to prevent the infection of COVID-19	644 (66)	334 (34)	978 (100)
Personal protective equipment (PPE) for COVID-19 consists of	Face shield/ goggles	964 (99)	14 (1)	978 (100)
	Gown	959 (98)	19 (2)	978 (100)
	Gloves	967 (99)	11 (1)	978 (100)
	N95 face mask	952 (97)	26 (3)	978 (100)
The correct method of donning PPE	Gathering the PPE, washing your hands, put on the gown, face mask, face shield/ goggles, gloves, enter the room	751 (77)	227 (23)	978 (100)
The correct method of removing PPE	Remove gloves, remove gown, exit the room, hand hygiene, remove face shield/goggles, remove face mask, dispose, wash hands again	421 (43)	557 (57)	978 (100)

**Table 5:** Study participants’ response pattern to questions related to attitude toward COVID-19

Statement	Number (%)					Total
	Strongly agree	Agree	Neutral	Disagree	Strongly disagree	
As a healthcare worker, I am at risk of being infected	762 (77.9)	180 (18.4)	30 (3.1)	3 (0.3)	3 (0.3)	978 (100)
I agree with the infection control measures to prevent the spread of the virus	823 (84.2)	130 (13.3)	20 (2)	2 (0.2)	3 (0.3)	978 (100)
I encourage physical distancing between healthcare workers	801 (82)	142 (14.5)	31 (3.2)	1 (0.1)	2 (0.2)	978 (100)
I believe that hospitalized patients infected with COVID-19 should be isolated with droplet precautions	630 (64.4)	188 (19.2)	103 (10.5)	48 (5)	9 (0.9)	978 (100)
I believe that the clinical environment has to be continuously disinfected	835 (85.4)	111 (11.3)	27 (2.8)	3 (0.3)	2 (0.2)	978 (100)
I would receive the vaccine once it is established	630 (64.4)	179 (18.3)	113 (11.6)	40 (4.1)	16 (1.6)	978 (100)

**Table 6:** Study participants’ response pattern to questions related to fear toward COVID-19

Statement	N (%)					Total
	Strongly agree	Agree	Neutral	Disagree	Strongly disagree	
I am afraid of spreading the infection to my family, which made me move out or isolate myself	388 (39.7)	290 (29.7)	170 (17.4)	105 (10.7)	25 (2.5)	978 (100)
I have anxiety (palpitations, sweating, or sense of impending danger or panic) whenever I think of COVID-19	111 (11.4)	139 (14.2)	186 (19)	351 (35.9)	191 (19.5)	978 (100)
My sleep and appetite are affected whenever I think of COVID-19	117 (12)	128 (13.1)	169 (17.3)	347 (35.5)	217 (22.1)	978 (100)
I am afraid of dying from COVID-19	124 (12.7)	147 (15)	211 (21.6)	299 (30.6)	197 (20.1)	978 (100)
I avoid reading updates concerning COVID-19 (from the news, social media) as it increases my anxiety	143 (14.6)	136 (13.9)	203 (20.8)	306 (31.3)	190 (19.4)	978 (100)
I often take leaves to prevent my attendance in the clinical setting	111 (11.3)	68 (6.9)	122 (12.5)	382 (39.1)	295 (30.2)	978 (100)
I avoid public settings (i.e. shopping centers, grocery stores) as much as possible	241 (24.6)	302 (30.9)	222 (22.7)	136 (13.9)	77 (7.9)	978 (100)
I fear a second wave of COVID-19	405 (41.4)	327 (33.4)	127 (13)	77 (7.9)	42 (4.3)	978 (100)

Sources of knowledge about COVID-19

**Figure 2:**
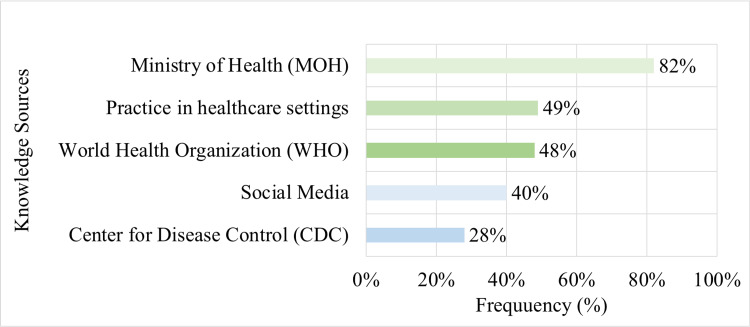
Knowledge sources about COVID-19

Participants were asked to choose one or more of the knowledge sources that helped them answer the questions of the knowledge section in the questionnaire. As shown below in Figure 2, 82% of participants attributed their knowledge to the Saudi Ministry of Health (MOH), 49% to their practice in the healthcare setting, and 48% to the World Health Organization (WHO).

Demographic variability of COVID-19 knowledge, attitude, and fear levels

Demographic Variability of COVID-19 Knowledge Level 

Out of all study participants (N = 978), knowledge level was high among 54.7% and low among 45.3%. A chi-square test of independence was performed to examine the relationship between the knowledge of respondents in relation to their demographic factors. The results showed that there was a significant difference in the knowledge level between sex, nationality, and role in healthcare. Females were shown to have a higher level of knowledge compared to males X2 (1, N = 978) = 8.9, p = .003, φc=.09. Non-Saudis had a higher level of knowledge compared to Saudis X2 (1, N = 978) = 11.7, p =. 00, φc=.11. Moreover, pharmacists and physicians had a higher level of knowledge compared to their counterparts X2 (5, N = 978) = 48.5, p =. 00, φc=.223 (Table 7).

**Table 7:** Demographic variability of COVID-19 knowledge, attitude, and fear levels

Demographic Information		N	Knowledge N (%)		P-Value	Attitude N (%)		P-Value	Fear N (%)		P-Value
			Low	High		Positive	Negative		Low	High	
Total	Total healthcare workers	978	443 (45.3)	535 (54.7)		527 (53.9)	451 (46.1)		446 (45.6)	532 (54.4)	
Sex	Female	617	257 (41.7)	360 (58.3)	0.003	322 (52.2)	295 (47.8)	0.164	265 (42.9)	352 (57.1)	0.029
	Male	361	186 (51.5)	175 (48.5)		205 (56.8)	156 (43.2)		181 (50.1)	180 (49.9)	
Age Group (Years)	20-29	414	197 (47.6)	217 (52.4)	0.145	239 (57.7)	175 (42.3)	0.263	220 (53.1)	194 (46.9)	0.000
	30-39	316	148 (46.8)	168 (53.2)		158 (50)	158 (50)		119 (37.7)	197 (62.3)	
	40-49	171	73 (42.7)	98 (57.3)		89 (52)	82 (48)		61 (35.7)	110 (64.3)	
	50-59	59	21 (35.6)	38 (64.4)		31 (52.5)	28 (47.5)		35 (59.3)	24 (40.7)	
	60-69	16	4 (25)	12 (75)		8 (50)	8 (50)		10 (62.5)	6 (37.5)	
	70-79	2	0 (0)	2 (100)		2 (100)	0 (0)		1 (50)	1 (50)	
Nationality	Non-Saudi	128	40 (31.3)	88 (68.8)	0.001	73 (57)	55 (43)	0.444	47 (36.7)	81 (63.3)	0.03
	Saudi	850	403 (47.4)	447 (52.6)		454 (53.4)	396 (46.6)		399 (46.9)	451 (53.1)	
Ethnicity	Black African descent	13	2 (15.4)	11 (84.6)	0.073	8 (61.5)	5 (38.5)	0.573	8 (61.5)	5 (38.5)	0.381
	Caucasian	22	7 (31.8)	15 (68.2)		14 (63.6)	8 (36.4)		13 (59.1)	9 (40.9)	
	Indian Asian	136	59 (43.4)	77 (56.6)		68 (50)	68 (50)		62 (45.6)	74 (54.4)	
	Middle Eastern	807	375 (46.5)	432 (53.5)		437 (54.2)	370 (45.8)		363 (45)	444 (55)	
Working Sector	Governmental	790	358 (45.3)	432 (54.7)	0.980	442 (55.9)	348 (44.1)	0.008	371 (47)	419 (53)	0.080
	Private	188	85 (45.2)	103 (54.8)		85 (45.2)	103 (54.8)		75 (39.9)	113 (60.1)	
Role in Healthcare	Dentist	63	28 (44.4)	35 (55.6)	0.000	28 (44.4)	35 (55.6)	0.038	30 (47.6)	33 (52.4)	0.000
	Health associate professionals	214	117 (54.7)	97 (45.3)		102 (47.7)	112 (52.3)		109 (50.9)	105 (49.1)	
	Health management and support personnel	143	91 (63.6)	52 (36.4)		71 (49.7)	72 (50.3)		64 (44.8)	79 (55.2)	
	Nurse	191	83 (43.5)	108 (56.5)		108 (56.5)	83 (43.5)		61 (31.9)	130 (68.1)	
	Pharmacist	61	16 (26.2)	45 (73.8)		35 (57.4)	26 (42.6)		19 (31.1)	42 (68.9)	
	Physician	306	108 (35.3)	198 (64.7)		183 (59.8)	123 (40.2)		163 (53.3)	143 (46.7)	
Health-Related Risk Factors for Severe COVID-19	Absent	619	267 (43.1)	352 (56.9)	0.074	334 (54)	285 (46)	0.952	290 (46.8)	329 (53.2)	0.304
	Present	359	176 (49)	183 (51)		193 (53.8)	166 (46.2)		156 (43.5)	203 (56.5)	

Demographic Variability of COVID-19 Attitude Level 

Among study participants, 53.9% had a positive attitude and 46.1% had a negative attitude toward COVID-19. Participants’ attitude was compared across the demographic variables using the chi-square test. There was a significant difference in the proportion of the working sector and role in healthcare. The government sector employees had a more positive attitude compared to the private sector employees X2 (1, N = 978) = 6.04, p =. 008, φc=.085. Regarding the role in healthcare, physicians displayed better positive attitude compared to their counterparts X2 (5, N = 978) = 11.78, p =. 038, φc=.110 (Table 7).

Demographic Variability of COVID-19 Fear Level

In terms of fear levels, 54.4% of participants were found to have high levels of fear toward COVID-19 while 45.6% were found to have low levels of fear. Healthcare workers' fear of COVID-19 was compared across the demographic’s variables using the chi-square test. There was a significant difference in the proportion of sex, age, nationality, and role in healthcare. Females tend to have higher fear levels compared to male participants X2 (1, N = 978) = 4.71, p =. 029, φc=.070. Healthcare workers aged between 30-49 have significantly higher fear levels compared to the other age groups (X2 (5, N = 978) = 30.65, p =.00, φc=.177). Nurses and pharmacists were shown to have higher fear levels compared to their counterparts X2 (5, N = 978) = 29.36, p =. 00, φc=.173 (Table 7).

Demographic variability of healthcare workers' COVID-19 risk level

Out of all study participants (N = 978), the risk level of COVID-19 was found to be high in 0.3% of participants, medium in 12.5%, and low in 87.2%. A chi-square test of independence was performed to examine the relation between the risk level of respondents in relation to their demographic factors. There was a significant difference in the proportion of sex, age, nationality, ethnicity, and health-related risk factors for severe COVID-19. Males had a higher risk level than females (X2 (2, N = 978) = 60.65, p =. 00, φc=.249). Participants aged between 70 and 79 years had a higher risk level compared to the other age groups (X2 (10, N = 978) = 453.09, p =. 00, φc=.481). Non-Saudis had a higher risk level than Saudis (X2 (2, N = 978) = 13.16, p =. 001, φc=.116). Black African participants had a higher risk level when compared with other ethnicities (X2 (6, N = 978) = 40.475, p =. 001, φc=.144). Participants who had health-related risk factors for severe COVID-19 had a higher risk level when compared with their counterparts (X2 (2, N = 978) = 124.767, p =. 000, φc=.357) (Table 8).

**Table 8:** Demographic variability of healthcare workers' COVID-19 risk level

Demographic information of Participants		N	Risk Level N (%)			P-Value
			Low	Medium	High	
Total	Total participants	978	853 (87.2)	122 (12.5)	3 (0.3)	
Sex	Female	617	577 (93)	40 (6.5)	0 (0)	.000
	Male	361	276 (76.5)	82 (22.7)	3 (0.8)	
Age groups (Years)	20-29	414	385 (93)	29 (7)	0 (0)	.000
	30-39	316	298 (94.3)	18 (5.7)	0 (0)	
	40-49	171	143 (83.6)	28 (16.4)	0 (0)	
	50-59	59	27 (45.8)	32(54.2)	0 (0)	
	60-69	16	0 (0)	14 (87.5)	2 (12.5)	
	70-79	2	0 (0)	1 (50)	1 (50)	
Nationality	Non-Saudi	128	99 (77.3)	28 (21.9)	1 (0.8)	.001
	Saudi	850	754 (88.7)	94 (11.1)	2 (0.2)	
Ethnicity	Black African descent	13	6 (46.2)	6 (46.2)	1 (7.7)	.001
	Caucasian	22	18 (81.8)	4 (18.2)	0 (0)	
	Indian Asian descent	136	124 (91.2)	12 (8.8)	0 (0)	
	Middle Eastern	807	705 (87.4)	100 (12.4)	2 (0.2)	
Working sector	Governmental	790	697 (88.2)	91 (11.5)	2 (0.3)	.092
	Private	188	156 (83)	31 (16.5)	1 (0.5)	
Role in health care	Dentist	63	62 (98.4)	1 (1.6)	0 (0)	0.050
	Health associate professionals	214	182 (85)	31 (14.5)	1 (0.5)	
	Health management and support personnel	143	122 (85.3)	21 (14.7)	0 (0)	
	Nurse	191	176 (92.1)	15 (7.9)	0 (0)	
	Pharmacist	61	55 (90.2)	6 (9.8)	0 (0)	
	Physician	306	256 (83.7)	48 (15.7)	2 (0.7)	
Health-Related Risk Factors for Severe COVID-19	Absent	619	596 (96.3)	23 (3.7)	0 (0)	.000
	Present	359	257 (71.6)	99 (27.6)	3 (0.8)	

Correlation between knowledge, attitude, fear, and risk scores

A Spearman's rank-order correlation was run to determine the relationship between knowledge, attitude, fear, and risk scores. There was a significant positive relationship between knowledge and attitude (rs = .188, p = .00). On the other hand, there was a significant negative relationship between knowledge and fear (rs = -.102, p = .00). Furthermore, there was a significant positive relationship between attitude and fear (rs = .079, p = .01). Risk scores did not show a significant relationship with knowledge, attitude, and fear scores. as shown in Table 9.

**Table 9:** Correlation between scores of knowledge, attitude, fear, and risk

		Knowledge Score	Attitude Score	Fear Score	Risk Score
Knowledge Score	Correlation Coefficient	NA	0.188	-0.102	-0.007
	P-value		0.000	0.001	0.837
Attitude Score	Correlation Coefficient	0.188	NA	0.079	0.013
	P-value	0.000		0.014	0.681
Fear Score	Correlation Coefficient	-0.102	0.079^*^	NA	-0.027
	P-value	0.001	0.014		0.394
Risk Score	Correlation Coefficient	-0.007	0.013	-0.027	NA
	P-value	0.837	0.681	0.394	

## Discussion

The rapid spread of COVID-19 has become one of the world’s major concerns, as of current times. The infection has affected the lives of millions, including an alarming number of HCWs. Similar, to the rest of the world, HCWs in KSA have also been affected by COVID-19. The purpose of this study was to quantify the level of knowledge, attitude, fear, and risk of COVID-19 among HCWs within the Eastern province of KSA. A total of 987 participants were surveyed, the majority of whom were females (63.1%), 20-39 years old (74.6%), of Saudi nationality, and worked as physicians.

In terms of knowledge, the majority of participants (54.7%) in the present study were found to have a high level of knowledge about COVID-19. Most of the participants scoring a high level of knowledge were consistent with previous studies done in Saudi Arabia that were conducted at different times of the pandemic, during 2020 [10,15]. It was also consistent with other studies from around the world, including a study done in Egypt [8], China [6], India [16], and Nigeria [17]. Considering, that the current study’s assessment of participants’ knowledge is more comprehensive, as it used 61 items in comparison to fewer questions in the previously mentioned studies; to be exact, 24, 9, 8, 8, 17, and 13 questions in the Egypt, India, Nigeria, and Saudi studies [8-10,15-17], respectively.

Most of the participants correctly answered the questions regarding COVID-19 virology, in which, 66% were aware of “SARS-CoV-2 being the causative agent of COVID-19” in comparison to a previous study done in KSA [10], where only 45% of its participants answered correctly. This finding is not consistent with studies done in Egypt [8] and India [16], where 100% and 99.7% of participants were able to answer correctly, respectively. This may be attributable to the fact that the aforementioned studies considered the participants' awareness of COVID-19 being caused by a viral agent, without specifying the viral nomenclature, sufficient enough to constitute a good level of virology knowledge.

Moreover, this study’s questionnaire included 15 items to assess the knowledge of the risk factors of COVID-19, where the majority of participants answered correctly. However, the studies done in Egypt [8] and previously in KSA [15], limited their questions regarding risk factors to one close-ended question only.

Furthermore, most of this study’s participants answered four out of five items correctly regarding the clinical features of COVID-19. The one item mostly answered incorrectly was “COVID-19 infection is a life-threatening disease and was associated with high mortality.” This raises the question of why HCWs, within the Eastern province, perceive COVID-19 to have high mortality when it has a much lower mortality rate compared to other pandemics [18]. This misconception can be attributed to the utilization of social media (40%) as a source of knowledge, which is filled with incorrect information.

The current study also tested the participant’s knowledge regarding the management of COVID-19 through four items, all of which were answered correctly by most participants. To illustrate, 78% of participants recognized the absence of a definitive cure for COVID-19. In contrast, in one Nigerian study, 90.8% of participants identified that there was “no effective cure for COVID-19 but palliative care can help most patients” [17]. This discrepancy can be attributed to the fact that the Nigerian study used leading statements to inquire about participants’ understanding of COVID-19 management.

Regarding the prevention of COVID-19, 99% correctly identified the role of face masks and social distancing in breaking the chain of infection [9]. When compared to another study conducted in KSA in April [10], only 68% agreed to the same statement. This difference can be explained by the intensified public awareness and education the government and healthcare facilities have advertised; in addition to the strict government regulations that have intensified throughout the past months.

Interestingly, higher overall knowledge scores were reported among females, which was unlike the study done in KSA in February, in which no difference between both genders in knowledge levels was reported [9]. In the present study, physicians were found to have higher knowledge, which is consistent with the study done in KSA in March through April [10]. This may be because physicians are exposed to a higher number of COVID-19 patients and are required to have a more specific understanding of the disease and its management.

The most cited source of knowledge in the present study was the Saudi Ministry of Health (MoH), which corresponds to the other study done in KSA in April, where 97% of participants reported MoH as their main source of information [10].

As for participants' attitude toward COVID-19, 53.9% of participants were found to have a positive attitude. These results are consistent with studies conducted in Egypt [8], India [16], and Nigeria [17]. However, a study done in KSA in April reported a negative attitude [19]. The difference between these two studies may be explained by the fact that during April, the number of reported positive COVID-19 cases in KSA was increasing and the course of the disease was still ambiguous, which may have impacted the attitude negatively.

In the current study, 77.9% of the participants strongly agreed to feeling at risk of infection as HCWs, which was similar to the study done in KSA in April. Moreover, 84.2% of the current study’s participants strongly agreed with the fact “infection control measures can prevent the spread of the virus”; which is higher than in the study done in KSA in April (71%) [19].

In this study, 64.4% of the HCWs strongly agreed to receive the vaccine once established. This is inconsistent with the other study done in KSA in December 2020, in which 33.27% were enrolled to receive or have already received the vaccine. However, this may be explained by the fact that the previous study was conducted during the first month of rollout and the number of registered HCWs to get vaccinated is continuously increasing beyond the conclusion of that study [20].

A more positive attitude was reported among HCWs in the governmental sector, where COVID-19 cases are expected to be higher than that of the private sector. Another statistically significant relationship was found between attitude and role in healthcare, with physicians having a more positive attitude as compared to their other counterparts. This perhaps can be attributed to their more scientific understanding of COVID-19 and its clinical course.

With regards to fear of COVID-19, 55.5% agreed to avoid public settings. This result is lower than that reported in the study done in KSA in April (95.7%). This may be explained by the fact that during April, a strict lockdown was implemented in KSA at the time [19].

In terms of participants' risk of COVID-19 infection, the majority had a low risk of infection. Remarkably, males were found to be more at risk of contracting COVID-19, which is inconsistent with a study done in Switzerland that assessed the risk of COVID-19 according to the seroprevalence of SARS-CoV-2 infection in HCWs. The study revealed no difference in seropositivity to anti-SARS-CoV-2 antibodies between males and females [21]. This may be due to the different parameters used to assess the risk of infection.

Moreover, HCWs aged between 70 and 79 years were reported to be more at risk of COVID-19, which is inconsistent with a study done in Switzerland, where being older than 50 years of age was associated with a lower seroprevalence in their population. This could be explained by the fact that at the age of 50 in Switzerland, HCWs were less represented in high-risk wards and units compared to younger HCWs [21].

Overall, in the present study, higher knowledge scores were found to be associated with a more positive attitude and lower fear scores toward COVID-19.

## Conclusions

The present study demonstrated significant sociodemographic variability among healthcare workers in the Eastern province with respect to their risk of contracting COVID-19 and their levels of knowledge, attitude, and fear toward the disease. This, in turn, can serve as a gateway for further research to understand why such sociodemographic variability exists among healthcare workers dealing with the same pandemic within the same geographical area. Moreover, this study, with its three newly developed and validated scales, can be used as a stepping-stone for further understanding HCWs’ knowledge, attitude, and fear levels throughout all provinces within KSA.
